# What is the prospect of indoleamine 2,3-dioxygenase 1 inhibition in cancer? Extrapolation from the past

**DOI:** 10.1186/s13046-021-01847-4

**Published:** 2021-02-08

**Authors:** Yu Yao, Heng Liang, Xin Fang, Shengnan Zhang, Zikang Xing, Lei Shi, Chunxiang Kuang, Barbara Seliger, Qing Yang

**Affiliations:** 1 State Key Laboratory of Genetic Engineering, Department of Biochemistry, School of Life Sciences, Fudan University, Songhu Road 2005, 200438 Shanghai, China; 2 Shanghai Key Lab of Chemical Assessment and Sustainability, School of Chemical Science and Engineering, Tongji University, 1239 Siping Road, 200092 Shanghai, China; 3 Institute of Medical Immunology, Martin Luther University Halle-Wittenberg, Magdeburger Straße 2, 06112 Halle (Saale), Germany

**Keywords:** Indoleamine 2,3-dioxygenase 1, Indoleamine 2,3-dioxygenase 1 inhibitor, Epacadostat, Tryptophan 2,3-dioxygenase, Cancer immunotherapy, Immune checkpoint

## Abstract

Indoleamine 2,3-dioxygenase 1 (IDO1), a monomeric heme-containing enzyme, catalyzes the first and rate-limiting step in the kynurenine pathway of tryptophan metabolism, which plays an important role in immunity and neuronal function. Its implication in different pathophysiologic processes including cancer and neurodegenerative diseases has inspired the development of IDO1 inhibitors in the past decades. However, the negative results of the phase III clinical trial of the would-be first-in-class IDO1 inhibitor (epacadostat) in combination with an anti-PD1 antibody (pembrolizumab) in patients with advanced malignant melanoma call for a better understanding of the role of IDO1 inhibition. In this review, the current status of the clinical development of IDO1 inhibitors will be introduced and the key pre-clinical and clinical data of epacadostat will be summarized. Moreover, based on the cautionary notes obtained from the clinical readout of epacadostat, strategies for the identification of reliable predictive biomarkers and pharmacodynamic markers as well as for the selection of the tumor types to be treated with IDO1inhibitors will be discussed.

## Background

With increasing recognition of evading immunological destruction as a hallmark of cancer acquired during its development[1], immunotherapy has emerged as a novel and important pillar of cancer treatment in addition to surgery, radiation, chemotherapy, and targeted therapy. Instead of directly killing cancer cells or suppressing the abnormal signal transduction within cancers, cancer immunotherapy aims to leverage the patient’s immune system to eliminate tumor cells by enhancing tumor immunity mediated by blocking immune inhibitory pathways and/or inhibitory cells in the tumor microenvironment (TME) or enhancing the specificity of anti-tumor immunity by inducing the expansion of T cells and antibodies directed to well-defined tumor antigens[2]. The launch of immune checkpoint inhibitors (ICPIs) including the first cytotoxic T-lymphocyte antigen 4 (CTLA-4) blocking antibody ipilimumab in 2011[3] and programmed death receptor 1 or ligand 1 (PD-1/PD-L1) blocking antibodies pembrolizumab and nivolumab in 2014[4, 5] represents the substantive progress in the field and boosts immunotherapy as a new standard therapy for many cancer types. To date, the US Food and Drug Administration has approved the use of PD-1/PD-L1 and CTLA-4 targeted therapies for the treatment of more than 15 cancers including melanoma, non-small cell lung cancer (NSCLC), small cell lung cancer, squamous cell carcinoma of the head and neck, renal cell carcinoma (RCC), hepatocellular carcinoma (HCC), classical Hodgkin lymphoma, urothelial carcinoma, colorectal cancer (CRC) and other cancer types[3–5]. However, limited response due to innate and acquired resistance as well as serious adverse effects especially life-threatening immune-related toxicities of anti-CTLA-4 and anti-PD-1/PD-L1 antibodies call for a need to identify other immunotherapy options targeting different mechanisms of immunosuppression in tumors that could be used alone or in combination with existing treatments. Currently over three thousand drugs directing against more than four hundred active targets are being investigated under different stages from preclinical to post-approval[6]. In this context indoleamine 2,3-dioxygenase 1 (IDO1) is one of the most studied target of cancer immunotherapies by virtue of its effects on immune suppression in the TME[6].

**Fig. 1:**
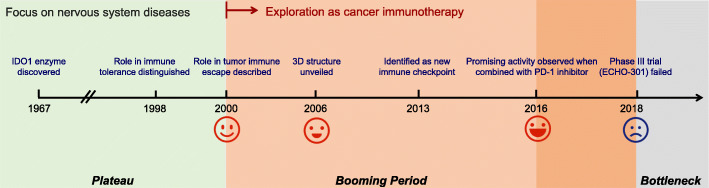
The roadmap of IDO1 inhibitor development. *IDO1* indoleamine 2,3-dioxygenase 1. *PD-1* programmed death receptor 1

IDO1, a monomeric heme-containing enzyme, is one of the three enzymes that catalyze the first and rate-limiting step in the kynurenine pathway (KP), leading to the degradation of the essential amino acid tryptophan (TRP) and generation of kynurenine (KYN) and other downstream metabolites, in addition to tryptophan 2,3-dioxygenase (TDO) and indoleamine 2,3-dioxygenase 2 (IDO2)[7, 8]. TRP is required for protein synthesis and TRP metabolites can act as neurotransmitters and signalling molecules. Thus, TRP and its metabolites play important roles in diverse physiological processes ranging from cell growth and survival to the coordination of responses to internal and external environmental changes[9]. IDO1 is expressed in various tissues, organs and cell types, catalyzing the conversion of a wide range of substrates including TRP and 5-hydroxytryptamine[10, 11]. TDO mainly exists in the liver and brain[12, 13], specifically catalyzing the conversion of TRP and some of its derivatives substituted in the 5- and 6-positions of the indole ring[14, 15]. TDO is thought to be the main modulator of TRP catabolism and is responsible for regulating systemic TRP levels[16], while IDO1 plays an important role in TRP catabolism under pathological conditions[17, 18]. The host-protective effect of IDO1 in parasitic infection has been first discovered in 1990s, which is attributable to a reduced availability of TRP in the inflammatory environment[19, 20]. In addition, as many KP metabolites are neuroactive, dysfunction of KP enzymes including IDO1 and TDO, often caused by inflammatory insults can trigger or facilitate diseases of the central nervous system, such as depression, Alzheimer’s disease, and Huntington’s disease[21–23]. IDO1 was first discovered to have a potent role in immune tolerance between a mother and fetus, since the inhibition of IDO1 in pregnant mice caused spontaneous immune rejection of allogeneic fetuses[24]. Later, the IDO1-mediated immune tolerance in tumors has been noted, experimental models suggest that IDO1 expression prevents rejection of tumor cells in immunogenic mice[25–30]. The imbalances in the level of TRP and KYN as well as the overexpression of IDO1 have been reported in many cancers[25–29, 31–47]. High IDO1 expression was associated with a decreased survival[48] and the extent of IDO1 overexpression depends on the tumor type and risk factors[49]. Three downstream effector pathways, including the general control nonderepressible 2 (GCN2), mammalian target of rapamycin (mTOR), and aryl hydrocarbon receptor (AhR) pathways, have been implicated in the biological responses to IDO1-mediated TRP depletion or KYN accumulation. In addition, some studies have shown that these effector pathways mediate immunosuppression in the TME[50–53]. TRP depletion promotes the accumulation of uncharged tRNA, resulting in a GCN2-dependent inhibition of protein synthesis that is accompanied by cell cycle arrest and irresponsiveness to immunological challenges[54]. TRP depletion leads to inhibition of the immunoregulatory kinases mTOR and protein kinase C, along with the induction of autophagy[55]. KYN accumulation promotes the translocation of AhR from the cytosol to nucleus, where it binds target genes and activates their transcription, inducing tolerogenic immune responses with the consequent benefit to tumor progression and migration[53, 56]. The exact contribution of each effector function to tumor immunosuppression remains to be determined. And it is likely that each pathway counts for much or less in different tumors although all of them cooperate synergistically in the development of immunosuppression in the TME. Based on these data, the inhibition of IDO1 has become an exciting approach as cancer immunotherapy and IDO1 inhibitors have been intensively investigated during the recent years. Multiple IDO1 inhibitors with different structural skeleton have been developed[57–67] and some have entered clinical development (Table 1). However, the further development of IDO1 inhibitors has experienced a significant setback due to the recent failure of the phase III trial (ECHO-301/KEYNOTE-252) of epacadostat (INCB024360), the would-be first-in-class IDO1 inhibitor, which had been anticipated to receive regulatory approval on the basis of promising results from initial studies (Fig. 1) [24, 50, 68–72].

**Table 1:** Summary of clinical trials of IDO1 inhibitors

	Number of initiated clinical trials (n)		
Compound/Phase	**Combination**	**Monotherapy**	**Total**
epacadostat	**54**	**5**	**59**
I or I/II	28	31	31
II	19	21	21
III	7	0	7
BMS-986205	**18**	**0**	**18**
I or I/II	7	0	7
II	7	0	7
III	4	0	4
indoximod	**12**	**2**	**14**
I or I/II	8	2	10
II	4	0	4
navoximod	**1**	**2**	
I	1	1	2
KHK-2455	**2**	**0**	**2**
I	2	0	2
PF-06840003	**0**	**1**	**1**
I	0	1	1
LY-3381916	**1**	**0**	**1**
I	1	0	1
DN1406131	**0**	**1**	**1**
I	0	1	1
Total	**88**	**10**	**98**

Several reviews discussing the biochemical properties and physiological functions of IDO1 and the therapeutic applications of IDO1 inhibition in cancer and other diseases have been published [8, 16, 73–75]. The aims of this article are, (i) to introduce the current status of clinical testing of IDO1 inhibitors (ii) to review representative data of epacadostat and (iii) to discuss some open questions raised from the failure of epacadostat in advanced malignant melanoma.

## IDO1 inhibitors under clinical development

Currently, as a focus of research and drug discovery efforts, there are at least eight small molecule IDO1 inhibitors under investigation in clinical trials indicated by the clinical trial registry website ClinicalTrials.gov. These include epacadostat (INCB024360; developer: Incyte), indoximod (D-1MT; developer: NewLink Genetics), navoximod (NLG919; developer: NewLink Genetics), BMS-986205 (Ono-7701/linrodostat; developer: Bristol-Myers Squibb/Ono), PF-06840003 (EOS-200,271; developer: Pfizer), KHK-2455 (developer: Kyowa Hakko Kirin), LY-3381916 (developer: Eli Lilly) and DN1406131 (developer: Jiangxi Qingfeng).

The first clinical trial of IDO1 inhibitor was started in 2007, which is the first-in-human (FIH) trial of indoxiomd with the purpose of assessing its safety and pharmacokinetics profile in patients with advanced solid tumors (NCT00567931). Since then to the cutoff date of 16 June 2020, a total of 98 clinical trials aiming to characterize the safety and efficacy profile of different IDO1 inhibitors have been initiated and the majority of them (88/98, 90 %) focuses on the exploration of treatment combinations (Table 1). Because one trial may have been tested in multiple cancer types, total 104 studies of 98 clinical trials have been carried out (Table 2). Seventy studies have been performed in 6 cancer types including lung cancer (16/70), head and neck cancer (12/70), gynecological tumors including ovarian and endometrial carcinoma (12/70), malignant melanoma (11/70), urothelial carcinoma (11/70) and brain tumors especially glioblastoma (8/70). Twenty studies did not specifically indicate the target cancer type or enrolled healthy volunteers only, and the rest studies have been performed in 15 cancer types including RCC, HCC and CRC (Table 2). In all studies, ICPIs, in particular anti-PD-1/PD-L1 antibodies were the most popular class selected as a combination agent, while others include chemotherapy, cancer vaccines, and other immunotherapies targeting CTLA-4, lymphocyte-activation gene 3, C-C motif chemokine receptor type 4 are also under investigation.

**Table 2:** Comparison of all initiated clinical trials by cancer type^a^

Cancer type	Number of initiated trials	Most advanced phase of trial
Lung cancer	16	III
Head and neck cancer	12	III
Gynecological tumors^b^	12	II
Melanoma	11	III
Urothelial carcinoma^c^	11	III
Brain tumors^d^	8	II
Other^e^	34	III

^a ^28 out of 98 trials that did not specifically indicate the target tumor type or enrolled healthy volunteers were excluded, and multiple cancar types could be tested in one trial. ^b ^Ovarian cancer, endometrial carcinoma, primary peritoneal carcinoma, and fallopian tube cancer were classified as gynecological tumors. ^c ^Bladder cancer was classified as urothelial carcinoma. ^d ^Glioma, glioblastoma, medulloblastoma, ependymoma were classified as brain tumors. ^e ^Others include breast cancer [5], CRC [4], RCC [4], gastric cancer and/or esophageal cancer [4], acute myeloid leukemia (AML) [3], pancreatic carcinoma [3], HCC [2], prostate cancer [2], diffuse large B-cell lymphoma (DLBCL) [1], gastrointestinal stromal tumor [1], myelodysplastic syndromes [1], nasopharyngeal carcinoma (NPC) [1], rectal cancer [1], sarcoma [1] and thymic carcinoma [1]

**Fig. 2:**
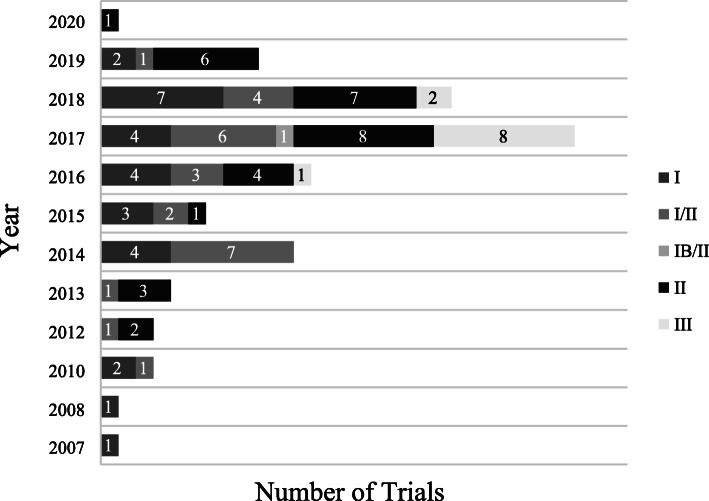
Comparison of all initiated clinical trials by start date^†^ and trial phase. †The calculation is according to the time of first posted date on clinicaltrials.gov

An analysis of the trial initiation (i.e. First posted date disclosed in ClinicalTrials.gov) over time (Fig. 2) shows that the clinical development of IDO1 inhibitors started from 2007, and the expansion significantly accelerated after 2014 with its peak in 2017. In 2017, the number of all initiated trials has grown to 27, representing a 125 % increase from 2016 to 2017 and the proportion of phase III trials has grown from 8 % (1/12) to 30 % (8/27) from 2016 to 2017. However, a rapid fade of interest in IDO1 inhibitors came up in 2018, the year that the failure of ECHO-301/KEYNOTE-252 was announced[72].

Epacadostat is an IDO1 selective inhibitor, which is the most advanced in the clinical development and would be supposed to be the first IDO1 inhibitor to get registration approval. Epacadostat showed preliminary promising anti-cancer activity in its early phase I/II trials (ECHO-202/KEYNOTE-037 and ECHO-204) when combined with anti-PD-1 drugs including pembrolizumab and nivolumab in patients with advanced malignant melanoma[76, 77]. However, its clinical value was not further validated in subsequently pivotal phase III trial (ECHO-301/KEYNOTE-252)[78] when the number of enrolled patients increased – the addition of epacadostat to pembrolizumab failed to improve progression-free survival (PFS) in patients with advanced malignant melanoma compared to pembrolizumab monotherapy.

Consequently, the unexpected setback of epacadostat has had a major impact on the entire field and tremendously slowed the pace of clinical development of all other IDO1-targeting compounds. In turn, total 27 trials involving 5 compounds, including epacadostat, BMS-986205, indoximod, PF-06840003 and LY-3381916, have been terminated, suspended or withdrawn and none of the other phase III programs of epacadostat is enrolling patients as originally planned. Just as every coin has its two sides, this failure also contributes to the understanding of the role of IDO1 inhibition and changes the clinical development program of inhibitors. Since the second half of 2018, seventeen new trials have been initiated and a large proportion of the clinical development is taking place in phase I/II, with only one agent-BMS-986205 proceeded to Phase III trial in bladder cancer (Fig. 2). Of note, the target patient populations in these new trials were narrowed down to certain tumor types, such as head and neck cancer, glioblastoma and bladder cancer. This may reflect that IDO1 is still regarded as a potential target to break the immune suppressive TME and restore the immune surveillance system. The critical challenges would be what we can learn from the failure and how to address the outstanding questions.

## The development path of epacadostat

In preclinical studies, epacadostat selectively and competitively inhibited the catalytic activity of IDO1 in multiple cell-based assays with little activity against IDO2 and TDO2[65, 79](Table 3). In co-cultures of human allogeneic lymphocytes with dendritic cells (DCs) or tumor cells, epacadostat promoted the growth of effector T cells and NK cells, reduced the conversion of naïve T cells to regulatory T cells (Tregs) and increased the number of CD86^high^ DC[79]. Along with these, administration of epacadostat to CT26 tumor-bearing mice reduced tumor growth with up to 57 % tumor growth control (TGC) in a dose-dependent and lymphocyte-dependent fashion and similarly decreased kynurenine levels in tumor tissues, plasma and draining lymph nodes ranging from 78–87 %[65]. As the most advanced IDO1 inhibitor, epacadostat was selected as a positive control in several preclinical studies of other IDO1 inhibitors in some animal models and showed similar IDO1 inhibitory activities and variable anti-tumor activities (Tables 3 and 4). Meanwhile, a synergy of INCB23843, an analogue of epacadostat, with anti-PD-L1 or anti-CTLA-4 antibodies, respectively, was investigated in B16 melanoma models. The combination with either anti-PD-L1 or anti-CTLA-4 antibody could suppress tumor growth more effectively than either agent alone, primarily through reactivation of anti-tumor immunity[80]. The combination of epacadostat with a MerTK inhibitor or an agonistic CD40 antibody also exhibited positive anti-tumor activities but epacadostat alone not[81, 82]. Furthermore, anti-PD-1 antibody, anti-CTLA-4 antibody and agonistic CD40 antibody therapies have been found to induce IDO1 expression[82, 83], which might be one reason for the limited response rate of these therapies. Therefore, the combination of IDO1 inhibitor with these therapies to defeat cancer has been paid high attention.

**Table 3:** Outline of IDO1 selective inhibitor epacadostat

**Assay**	**Enzyme source/cell**			**IC**_**50**_ **(nM)**	**Ref**
**Enzymatic**	His-tagged human IDO1			71.8	[79]
**Cellular**	CT26 colon carcinoma cells			76	[65]
	Pan02 pancreatic adenocarcinoma cells			27	[65]
	293/MSR-human IDO1 ^a^			15.0	[79]
	293/MSR-mouse IDO1 ^a^			52.4	[79]
	293/MSR-mouse IDO2 ^a^			> 5000	[79]
	293/MSR-human TDO ^a^			> 10,000	[79]
	Human dendritic cells			12.7	[79]
	Hela human cervical carcinoma cells			7.1	[79]
	SKOV-3 ovarian cancer cell			15.3	[84]
**Type of inhibition**	**Ref**	**Ki**	**Ref**	**Binding sites**	**Ref**
Competitive ^b^	[85]	1 µM ^b^	[85]	Heme iron ^c^	[86]

^a^ HEK293/MSR cells were transfected with full-length human or mouse IDO1 or mouse IDO2 cDNA, or a human TDO expression vector; ^b^ Type of inhibition and Ki of 4-amino-1,2,5-oxadiazole-3-carboximidamide which shares same skeleton as epacadostat; ^c^ Epacadostat coordinates to the heme iron of human IDO1; *IC*_*50*_ the concentration that exerts 50 % of the maximal inhibition effect on IDO1

**Table 4:** Anti-tumor performance of epacadostat in preclinical studies

Mouse model	Tumor growth reduction	Impact on immune function	Ref
CT26 colon carcinoma	34 % ^a^ at 30 mg/kg BID 57 % ^a^ at 100 mg/kg BID	Activity of tumor infiltrating lymphocytes increased	[65]
PAN02 pancreatic adenocarcinoma	37 % ^a^ at 25 mg/kg BID 57 % ^a^ at 100 mg/kg BID	-	[79]
CT26 colon carcinoma	47.5 % ^b^ at 100 mg/kg BID	-	[87]
Lewis lung carcinoma	51 % ^c^ at 80 mg/kg daily	Intratumoural ratio of CD4 effector T cells to Tregs elevated	[88]
B16F10 melanoma	50 % ^c^ at 80 mg/kg daily	Intratumoural ratio of CD4 effector T cells to Tregs elevated	[88]
Lewis lung carcinoma	68 % ^d^ at 60 mg/kg daily	Intratumoural ratio of CD4 effector T cells to Tregs elevated	[89]
CT26 colon carcinoma	23.4 % ^b^ at 100 mg/kg BID	-	[90]
RIL-175 hepatocellular carcinoma	Significant tumor reduction when combined with anti-PD-1, not significant at 300 mg/kg as single agent	-	[83]
MMTV-Neu breast cancer	Tumor growth inhibition enhanced when combined with lapatinib and BMS777607 (MerTK inhibitor), not significant reduction at 20 mg/kg as single agent	-	[81]
B16F10 melanoma	Significant tumor reduction when combined with agonistic CD40 antibody, not significant reduction at 100 mg/kg as single agent	Percentage of CD8^+^ CD107a^+^ effector T cells in tumor increased	[82]
B16SIY melanoma	Complete responders found when combined with αCTLA-4 (3/16) and αPD-L1 (2/15) ^e^	Frequency of tumor-reactive T cells in tumor-draining lymph nodes and spleen not substantially increased	[80]

Dosage regimens and administration methods varied across studies listed in table

^a ^Tumor growth control (TGC). ^b^ Tumor growth inhibition. ^c^ Tumor weight reduction. ^d^ Tumor volume reduction. ^e^ INCB23843 (the analogue of epacadostat) was used in this study. *BID* twice daily. *Tregs* regulatory T cells

With the support of the results generated from the above summarized preclinical data, the activity of epacadostat as single agent was evaluated in a FIH phase I trial (NCT01195311) and subsequently in a phase II trial (NCT01685255). In the FIH phase I trial, the safety, pharmacokinetics (PK), pharmacodynamics (PD) and preliminary anti-tumor efficacy profile of epacadostat were characterized in 52 patients with advanced solid malignancies. The results showed that the safety profile of epacadostat was manageable and it can be generally well tolerated at doses of up to 700 mg twice daily (BID). The PK of epacadostat was characterized by a time of maximum concentration at around 2 hours, a dose-independently biphasic disposition with an apparent terminal-phase disposition half-life of 2.9 hours and a relatively longer effective half-life of 4 to 6 hours in the context of systemic accumulation following BID dosing[91]. Regarding PD assessments, in addition to the direct evaluation of the plasma concentration of TRP and KYN in patients at different time points, the levels of TRP and KYN in patients blood pretreated with interferon gamma (IFN-γ) or lipopolysaccharide (LPS) were also determined. IFN-γ or LPS was used to induce IDO1 expression and minimize the confounding effect of TDO. Tracking KYN levels in plasma has been considered as a sufficient method for assessing IDO1 activity and selected as a valuable pharmacodynamic marker in the clinical studies of epacadostat[65]. The percentage of IDO1 inhibition was finally defined as the reduction in plasma KYN levels from the predose to the postdose value. It was found that epacadostat could reduce KYN levels at doses ≥ 100 mg BID and a 90 % inhibition was achieved when the plasma concentration of epacadostat was greater than 500 nmol/L[91]. Epacadostat’s on-target potency, i.e., its concentration exerting 50 % of the maximal inhition effect (IC_50_) against IDO1, is important for dose selection but complicated by the bioconversion of TRP to KYN catalyzed by both IDO1 and TDO. *In vitro* and *ex vivo*, the IC_50_ was estimated following the selective induction of IDO1, rendering the TDO activity relatively insignificant; however, it was desirable to determine the *in vivo* IC_50_ without inducing IDO1. Based on the data from the FIH trial, a mechanism-based population PD model was developed, and epacadostat *in vivo* IC_50_ was estimated to be ~ 70 nM, consistent with the *ex vivo* value independently determined. And this model also suggests that approximately 60 % of TRP to KYN bioconversion was attributed to IDO1 in the cancer patients at baseline, the mean concentration of plasma KYN was 2–3 % of that of TRP, and no significant changes in TRP concentrations were observed before or during epacadostat treatment[92]. Based on PK and PD data, 300 mg BID was selected as the recommended phase II monotherapy dose to provide sufficient drug exposure that would inhibit > 90 % of IDO1 activity[91]. The therapeutic efficacy profile from this FIH trial suggested a limited anti-tumor activity of epacadostat in human, only stable disease (SD), which is one of response category[93], was observed as the best overall response in 18 of 52 patients, and SD lasting ≥ 16 weeks was seen in 7 patients [91].

The following phase II trial aiming to compare the efficacy of epacadostat versus tamoxifen as therapy for biochemically recurrent (CA-125 relapse)–only epithelial ovarian cancer reported similar results. Epacadostat was generally well tolerated, but its efficacy was not satisfactory enough, i.e. no significant difference in efficacy was found between epacadostat and tamoxifen, and thus the trial was terminated accordingly[94]. These results are not that unexpected, since multiple mechanisms are involved in the evasion of tumors from immune surveillance and IDO1 inhibition strategy alone may be insufficient. Given that the synergy of IDO1 inhibitor and ICPIs was confirmed in B16 melanoma models[80], several phase I/II clinical trials were then initiated to investigate epacadostat in combination with ipilimumab[95] (NCT01604889), pembrolizumab[76] (NCT02178722) and nivolumab[77] (NCT02327078) for the treatment of unresectable or metastatic malignant melanoma.

In the phase I/II trial investigating epacadostat in combination with ipilimumab (NCT01604889), epacadostat ≤ 50 mg BID was demonstrated to bear clinical and pharmacologic activity and was generally well tolerated in patients with advanced melanoma[95]. Objective responses were observed in 9 of 39 immunotherapy-naive patients (23 %), while no objective response was seen in the rest 11 patients who previously received immunotherapy although 3 of them (27 %) had SD. Using the same method and definition of IDO1 inhibition as in the previous FIH trial, PD data from this trial showed that a dose-dependent inhibition of IDO1 by epacadostat and the average IDO1 inhibiton exceeded 50 % at doses of 25 mg BID and 50 mg BID[95]. These results were generally consistent with previously reported data in patients with advanced solid malignancies[91]. The emerging success of anti-PD-1 antibodies for the treatment melanoma led to an early termination of this trial (NCT01604889, only phase I part was conducted) and shift more attention to exploring epacadostat in combination with anti-PD-1 antibodies.

ECHO-202/ KEYNOTE-037 (NCT 02178722) was a phase I/II trial aiming to evaluate epacadostat plus pembrolizumab in patients with advanced solid tumors. The results from its phase I part showed that the combination regimen of epacadostat and pembrolizumab was well tolerated and a maximum tolerated dose (MTD) of epacadostat did not reach within pre-defined dose levels up to 300 mg BID; ≥50 % time-averaged IDO1 inhibition (i.e. the level of pharmacodynamic activity associated with inhibition of tumor growth seen in nonclinical models) determined with the above mentioned PD model was yielded in all patients treated with epacadostat 100 mg BID or 300 mg BID; objective responses were observed in 25 of total 62 patients (42 %) and 12 of 22 patients with advanced malignant melanoma (55 %)[76]. The phase II trial of epacadostat in combination with nivolumab, i.e. ECHO-204 (NCT02327078) having similar study design and patient population demonstrated consistently promising anti-tumor activity with objective response at 62 % and tolerable safety profile in 50 patients with advanced malignant melanoma[77]. On the basis of these results, epacadostat 100 mg BID was selected to combine with anti-PD-1 antibodies for additional investigation in phase III trials including ECHO-301/KEYNOTE-252 (NCT02752074)[78].

A non-randomized trial using a small number of patients is prone to biased selection that may confound the result and be misleading. Thus, a further confirmation with a randomized phase II trial as gatekeeper before entering into pivotal phase III trial might be necessary. However, regardless of the lack of evidence from at least one randomized, active controlled (anti-PD-1 antibody monotherapy) phase II trial as a validation step, the phase III trial ECHO-301/KEYNOTE-252 was initiated to further clarify the clinical benefit of epacadostat plus pembrolizumab compared to pembrolizumab alone in patients with advanced malignant melanoma previously untreated with a ICPI using PFS as primary endpoint. In this trial, the testing of IDO1 expression status by use of in situ hybridization RNA scope technology was retrospectively completed after patients were enrolled, but was not taken into account in the treatment assignment or primary analysis of the endpoints. Among all enrolled 706 patients, IDO1 expression was positive in 451 (90 %) of 502 patients with evaluable tumor specimens, and the IDO1 expression positivity was determined as higher than 1 % of tumor or immune cells expressed IDO1. The positive expression of IDO1 in tumor cells, intratumoral immune cells and both kinds of cells was found in 393 of 502 samples (78 %), 422 of 472 samples (89 %) and 364 (77 %) of 472 samples, respectively. As of data cutoff, surprisingly, the trial failed to prove the clinical value of epacadostat in terms of PFS, overall survival (OS), and objective response with a median PFS of 4.7 months (95 % CI 2.9–6.8) in the epacadostat plus pembrolizumab group and 4.9 months (2.9–6.8) in the placebo plus pembrolizumab group (HR 1.00, one-sided P = 0.52). Furthermore, the absence of a significant PFS benefit for epacadostat was evident in all prespecified and post-hoc subgroups examined including IDO1 status (Table 5).

**Table 5:** Profile of representative clinical trials of epacadostat

Treatment	Identifier/Trial name	Phase	Patients	Key results	Ref
Epacadostat	NCT01195311	I	52	1. MTD not established with dosing up to 700 mg BID 2. Near maximal changes observed at doses of ≥ 100 mg BID with > 80–90 % IDO1 inhibition achieved 3. Stable disease lasting ≥ 16 weeks observed in 7 patients 4. 300 mg BID selected as the recommended phase II monotherapy dose 5. PD modelling established	[91, 92]
Epacadostat	NCT01685255	I/II	42	1. Trial terminated due to slow accrual and lack of evidence of superiority 2. No significant difference in efficacy observed compared to tamoxifen in biochemical-only relapse ovarian cancer 3. IDO1 expression observed in 94 % of archival tumor samples	[94]
Epacadostat + ipilimumab	NCT01604889	I/II	50	1. Doses ≤ 50 mg BID generally well tolerated when combined with ipilimumab 2. IDO1 inhibition achieved with epacadostat doses ≥ 25 mg BID	[95]^a^
Epacadostat + pembrolizumab	NCT02178722 ECHO-202/ KEYNOTE-037	I/II	62	1. 100 mg BID plus pembrolizumab recommended for phase II evaluation 2. 12 (55 %) of 22 patients with melanoma achieved objective response	[76]^b^
Epacadostat + nivolumab	NCT02327078 ECHO-204	I/II	50	1. 100 mg BID plus nivolumab recommended for phase III evaluation 2. 31 (62 %) of 50 patients with melanoma achieved objective disease	[77]^c^
Epacadostat + pembrolizumab	NCT02752074 ECHO-301/KEYNOTE-252	III	706	1. No significant difference in PFS found between the treatment groups (median 4.7 months for epacadostat plus pembrolizumab vs. 4.9 months for pembrolizumab alone; [HR] 1.00; one-sided p = 0.52) 2. No significant difference in PFS found between the treatment groups in IDO1-positive patient	[78]

^a ^The trial was originally designed as a phase I/II trial and later suspended further patient enrollment in phase I dose-expansion and phase II. Only data from phase 1 dose-escalation portion of the trial was reported. ^b^ Data from phase I part was reported here. ^c^ Only data from phase II part was reported. *MTD* maximal tolerated dose. *BID* twice daily. *PFS* progression-free survival. *HR* hazard ratio. *CI* confidential interval

The failure of ECHO-301 has been discussed in terms of the sufficiency of the dose of epacadostat and drug combination selection[96, 97]. Furthermore, the signaling activity of IDO1 besides its catalytic activity may be attributed to another reason for the failure[97]. It has been described that the immunoregulatory activity of IDO1 is related to its signaling activity in addition to its catalytic activity[98, 99]. It could be the case and would offer a hypothesis that some IDO1 inhibitors, which have been designed for blocking the catalytic activity of IDO1 only, may not in reality block its signaling activity and thus could not be sufficient to “neutralize” the effects of IDO1. Although the failure of ECHO-301 trial upset the whole field as the benchmarker of IDO1 inhibitors, all preclinical and clinical data from epacadostat discussed above is like a treasure for the successors to excavate and get potential inspiration for future direction.

## Future directions

Despite the failure of epacadostat to demonstrate clinical benefits in the pivotal phase III trial, there is much to be learned from the available data, which is likely to ultimately benefit the field. It is too arbitrary and premature to deny the future of the entire field as the result of a single trial failure in the context of some reasonable doubt raised accordingly.

### Which biomarker should be used for IDO1 selective inhibitor therapy?

The term biomarker is commonly understood as referring to a characteristic that is measured as an indicator of normal biologic processes, pathogenic processes, or responses to an exposure or intervention, including therapeutic interventions[100]. At present, no biomarker has been used for patients’ selection in IDO1 inhibitors clinical trials although the IDO1 expression status has being explored in some trials to assess its correlation with response.

In a way, IDO1 expression is a kind of “straightforward choice” for the selection of IDO inhibitor predictive biomarker. A series of evidence could support this choice. As a part of a tumor immune escape mechanism, IDO1 overexpression has been described in numerous human cancer types not only in tumor lesions by tumor cells and other components of the TME like endothelial cells, myeloid derived suppressor cells (MDSCs), DCs, but also in tumor draining lymph nodes[30, 101, 102]. Several lines of studies have demonstrated that the overexpression of IDO1 in either tumor cells or in other cells within TME is associated with a more aggressive cancer phenotype, advanced disease stage and worse clinical outcome of various cancers including ovarian carcinoma, CRC, endometrial cancer, melanoma, cervical cancer, glioblastoma, lung adenocarcinoma, diffuse large B-cell lymphoma (DLBCL)[25–29, 31–36, 38]. Furthermore, a recent meta-analysis of 2706 patients from 24 articles found that high IDO1 expression is correlated with poor clinical outcomes in all cancers[37]. However, the results from the phase III trial (ECHO-301/KEYNOTE-252) did not show significant PFS benefit with epacadostat plus pembrolizumab therapy in overall population as well as the post-hoc IDO1-positive subgroup, despite 90 % patients were IDO1-positive[78]. This finding suggests that the expression of IDO1 in both tumor cells or immune cells is an important - but not a definitive - predictive biomarker of response to IDO1 inhibition.

Some reports give clues to explain the indefiniteness of IDO1 expression being a biomarker. First, there is an uncertainty of an exact correlation between IDO1 expression and tumor progression or patients’ outcome. Despite the positive correlation between high IDO1 expression and advanced cancer stage or poor patient prognosis[25–29, 31–36, 38], the negative correlation is found in patients with RCC[103] and contradictory correlations are documented in patients with HCC[104, 105]. Using Kaplan-Meier survival analyses, it has been found that no statistically significant relationship between OS, post-progression survival and distant-metastasis-free survival rates and tumour cell-derived IDO1 expression exists in patients with various types of cancers, such as lung, ovarian, breast or gastric cancer[88].

In addition, the method used to assess IDO1 expression was different from one study to another. Furthermore, the definition and grade of IDO1-positive tumors, as well as the nature of the IDO1-expressing cells were not always consistent. In the phase II trial (ECHO-202) and the phase III trial (ECHO-301/KEYNOTE-252), the methods used to assess IDO1 expression were the same (in situ hybridization using RNAscope technology), while the nature of expressing cells and threshold of positivity were different[76, 78]. In the phase II trial, only tumor-infiltrated immune cells were tested for IDO1 expression, and a histoscore ≥ 5 was used as an arbitrary cutoff for the IDO1-positive status. In the phase III trial, IDO1 positivity was defined as expression in more than 1 % of tumor or intra-tumoral immune cells.

Furthermore, the heterogeneity of IDO1 expression in tumor tissues also affect the detection results of IDO1 expression. Theate et al. assessed IDO1 expression by immunohistochemistry on tissue microarrays in 15 common solid tumor types with about 17–60 samples of each and found that the IDO1 expression could be identified in total 383 of 624 samples (61 %), but was limited to a small fraction of cells, and the proportion of IDO1-expressing cells widely varies according to the tumor type[101]. Among these 15 tumor types, cervical carcinoma (17/17, 100 %), endometrial carcinoma (45/48, 94 %) and bladder urothelial carcinoma (15/16, 94 %) most frequently expressed IDO1, followed by RCC (43/53, 81 %), NSCLC (41/51, 80 %), CRC (46/59, 78 %), head and neck cancer (14/24, 58 %) and melanoma (32/60, 53 %) while most glioblastoma (5/60, 8 %) were negative. Of note, the staining patterns of IDO1 varied widely according to the tumor type. For example, vascular staining was predominated in RCC (36/43, 84 %), which was consistent with previous report[103], whereas DC-like staining and tumor cell staining were predominated in CRC (45/46, 98 %) and endometrial carcinomas (39/45, 87 %), respectively[101]. It can be found that IDO1 is mainly expressed in the vasculature in RCC lesions rather than in tumor cells as described for other cancers. This variation may contribute to the contradictory phenomenon that the expression of IDO1 correlates with long survival of patients with RCC. Furthermore, in the phase III trial (ECHO-301/KEYNOTE-252), the high proportion of IDO1-positive samples was mainly attributable to the staining of immune cells rather than tumor cells[78]. Establishing a standard and uniform procedure of IDO1 expression assessment, for instance, a deep semi supervised generative learning for automated tumor proportion scoring which has been applied to standardization of PD-L1 expression and status[106], is required so that the extrapolation from non-clinical to clinical and from small scale to large scale may be more reliable.

Based on the clinical trial information, PD-L1 expression, tumor mutation burden (TMB), microsatellite instability-high (MSI-H) and mismatch-repair deficiency (MMR) have been identified as predictive biomarkers for anti-PD-1/PD-L1 antibody therapies[107–111]. Although biomarkers of IDO1 inhibitors gleaned from clinical trials is awaited, we here suggest that leveraging existing databases, such as The Cancer Genome Atlas, to figure out the molecular characteristics associated with the effect of IDO1 on prognosis might be a further approach. In fact, by this way, we have identified that the expression of 43 key genes is a better biomarker than the patient category regarding to the efficacy of IDO1 inhibitors (unpublished data).

### Whether plasma KYN concentration is an efficient pharmacodynamic marker?

Due to the lack of assays, which allow to directly determine the activity of the IDO1 enzyme, measuring the changes of the TRP, KYN, or KYN/TRP ratio has been selected in different studies as a surrogate indicator for activity evaluation. Ideally, these measurements should be tested in tumor tissues, which can provide intuitive evidence of IDO1 inhibition. Nevertheless, the majority of current available preclinical data on IDO1 activity are derived from serum. In clinical trials of epacadostat, IDO1 activity was determined by only measuring the inhibition of the plasma KYN concentration. However, the depletion of TRP is not always synchronized with accumulation of KYN in human cancers (Table 6). In fact, TRP depletion is more sensitive to disease condition than Kyn accumulation, not only in cancers, but also in other diseases such as Alzheimer’s disease and atherosclerosis[112–114]. TRP deprivation is significantly correlated with clinical outcomes like the quality of life in CRC, survival in malignant melanoma and advanced disease stage in lung cancer[39–41] while the KYN generation has a prognostic significance in adult T-cell leukemia/lymphoma (ATL)[42]. Thus, it will be more informative to use of TRP, KYN concentrations and the KYN/TRP ratio for the IDO1 activity assessment and the dose selection from PD perspective.

**Table 6:** TRP and KYN changes in human cancers^a^

Tumor type	No. of patients	Source	TRP	KYN	KYN/TRP ratio	Ref
CRC	66	serum	↓	−	↑	[39]
Melanoma	87	serum	↓	−	↑	[40]
Lung	123	serum	↓	↑	↑	[41]
Lung	36	serum	↓	−	↑	[43]
Ovarian carcinoma	20	serum	↓	−	↑	[44]
Gynecologic cancers^b^	109	serum	−	↑	↑	[45]
CRC	69	serum	?	↑	?	[116]
Lung	33	serum	?	?	↑	[46]
ATL	96	serum	−	↑	↑	[42]
Glioblastoma	10	serum	↓	↓^c^	−	[47]
Cervical cancer	27	tumor	↓	↑	↑	[117]

^a^ symbols used: ↑ increase, ↓ decrease, - no change, ? not reported (defined as statistically significant change). ^b^ include endometrial, ovarian, and vulvar cancer. ^c^ KYN serum level was significantly decreased in glioblastoma patients compared to healthy controls

Badawy et al. suggested that IDO1 induction is not the only determinant of the increase in the plasma KYN/TRP ratio *in vivo*[115]. Other determinants are liver TDO activity, increased flux of Trp through TDO and KP enzymes influencing KYN, especially kynurenine 3-monooxygenase, kynureninase and to a lesser extent kynurenine aminotransferase. Measurements of these additional determinants has been suggested to be able to contribute to a better understanding of the TRP status and IDO1 activity in IDO1 involved cancer and other diseases such as schizophrenia[115]. Furthermore, KYN/TRP ratio has also been known to be positively correlated with the concentrations of neopterin[40, 43], IL-6 as well as sIL-2Rα[44]. In summary, we suggest to determine the parameters TRP, KYN and the ratio of both in tumor and peripheral blood as primary PD marker in small studies for dose selection, before moving forward to large scale studies, and try to collect more information on other determinants of the KYN/TRP ratio if possible.

### What is the most possible candidate tumor type?

Until now, lung cancer is the most-studied tumor type in clinical trials of IDO1 inhibitors (Table 2). However, the reason, why so much attention has been paid to lung cancer, is not sufficient. Most clinical trials of IDO1 inhibitors have been carried out to test the anti-PD-1 antibody combination in a tumor type where anti-PD-1 antibody alone has activity.

There are 17 new trials initiated after the failure of ECHO-301. The newest ones are a phase II trial (NCT04231864) to evaluate epacadosat plus durvalumab, a anti-PD-L1 antibody, for treatment of advanced Epstein-Barr virus (EBV) positive nasopharyngeal carcinoma (NPC) and a phase III trial (NCT03661320) to assess IDO1 inhibitor (BMS-986205) in combination with nivolumab and chemotherapy (gemcitabine and cisplatin) as neoadjuvant treatment in patients with muscle-invasive bladder cancer. Despite clinical trials in bladder cancer have been initiated before, the newly initiated phase II trial (NCT04231864) is the first trial of IDO1 inhibitor in NPC. *In vitro*, exposure to the milieu created by IDO1-positive NPC cell line CNE2 cell impaired the lymphocyte cytotoxicity[118]. IDO1 activity defined as KYN/TRP ratio is significantly higher in patients with NPC and could be a relevant marker for advanced NPC progression[119]. Meanwhile, IDO1 is expressed by either tumor cells or tumor-associated immune cells in 99 % or 94 % patients with NPC using 1 % cutoff value of immunohistochemistry and negatively associated with OS[120]. Furthermore, an immune checkpoint-based signature classifier, in which IDO1 is one out of five features was significantly correlated with the survival in patients’ with high EBV-DNA load[120]. Furthermore, the upregulation of PD-L1 expression induced by the latent membrane protein 1 and IFN-γ was detected in EBV-positive NPC cell lines[121]. Together, these findings provide solid evidence that IDO1 is involved in immune evasion of NPC, which forms the rationale for pursuing clinical trials.

Recently, we have reported that the expression of both IDO1 and TDO was associated with advanced stage of disease and poor prognosis in patients with glioblastoma, which attributed to Kyn-AhR-AQP signaling pathway and an IDO1/TDO (IDO1 and TDO) dual inhibitor could exert anti-glioma effects in GL261 orthotopic glioma mice[122]. TDO has also been regarded as a logical candidate to mediate resistance to IDO1-selective inhibition besides its direct roles in cancer, the expression of TDO was detected in lots of cancer types such as HCC, glioblastoma, bladder carcinoma, pancreatic carcinoma, colon carcinoma, melanoma and breast cancer[56, 123–125]. About one-third of cancer cell lines harboring high levels of KYN have simultaneous IDO1 and TDO expression, while the others are driven by either IDO1 or TDO[126].

In conclusion, aberrant activation of KP as well as a solid correlation between high expression of IDO1 or IDO1/TDO and tumor progression or poor patients’ outcome should be kept in mind when selecting the candidate tumor type for clinical trials of IDO1 inhibitors.

## Conclusions

Extensive evidence supports the notion that an elevated TRP catabolism contributes to immune regulation that promotes tumor growth and therapy resistance. Targeting this biochemical pathway in patients with cancer offers promise. Preclinical and preliminary clinical data demonstrate evidence to anti-tumor activity of IDO1 inhibitors. However, setbacks in a recent clinical trial suggested an urgent need for more research to better understand what is the exact mechanism responsible for the immunosuppresion of IDO1 in cancer, how the TRP catabolism contributes to immune regulation mechanisms in the TME, what is the role of KYN and TRP in different cancer types, and to identify biomarkers to select patients that ultimately benefit from this treatment.

## Data Availability

All the data and materials are available upon reasonable request from the corresponding author.

## Abbreviations

AhR: Aryl hydrocarbon receptor
AML: Acute myeloid leukemia
ATL: Adult T-cell leukemia/lymphoma
BID: Twice daily
CRC: Colorectal cancer
CTLA-4: Cytotoxic T-lymphocyte antigen 4
DC: Dendritic cell
DLBCL: Diffuse large B-cell lymphoma
EBV: Epstein-Barr virus
FIH: First-in-human
GCN2: Nonderepressible 2
HCC: Hepatocellular carcinoma
ICPI: Immune checkpoint inhibitor
IDO1: Indoleamine 2,3-dioxygenase 1
IFN-γ: Interferon gamma
IDO2: Indoleamine 2,3- dioxygenase 2
KP: Kynurenine pathway
KYN: Kynurenine
LPS: Lipopolysaccharide
mTOR: Mammalian target of rapamycin
NK: Natural killer
NPC: Nasopharyngeal carcinoma
NSCLC: Non-small cell lung cancer
OS: Overall survival
PD: Pharmacodynamics
PD-1: Programmed death receptor 1
PD-L1: Programmed death ligand 1
PFS: Progression-free survival
PK: Pharmacokinetics
RCC: Renal cell carcinoma
SD: Stable disease
TDO: Tryptophan 2,3 dioxygenase
TME: Tumor microenvironment
TGC: Tumor growth control
Treg: Regulatory T cell
TRP: Tryptophan
